# Dietary Restriction Depends on Nutrient Composition to Extend Chronological Lifespan in Budding Yeast *Saccharomyces cerevisiae*

**DOI:** 10.1371/journal.pone.0064448

**Published:** 2013-05-17

**Authors:** Ziyun Wu, Shao Quan Liu, Dejian Huang

**Affiliations:** Food Science and Technology Programme, Department of Chemistry, National University of Singapore, Singapore, Republic of Singapore; Lancaster University, United Kingdom

## Abstract

The traditional view on dietary restriction has been challenged with regard to extending lifespan of the fruit fly *Drosophila melanogaster*. This is because studies have shown that changing the balance of dietary components without reduction of dietary intake can increase lifespan, suggesting that nutrient composition other than dietary restriction play a pivotal role in regulation of longevity. However, this opinion has not been reflected in yeast aging studies. Inspired by this new finding, response surface methodology was applied to evaluate the relationships between nutrients (glucose, amino acids and yeast nitrogen base) and lifespan as well as biomass production in four *Saccharomyces cerevisiae* strains (wild-type BY4742, *sch9*Δ, *tor1*Δ, and *sir2*Δ mutants) using a high throughput screening assay. Our results indicate that lifespan extension by a typical dietary restriction regime was dependent on the nutrients in media and that nutrient composition was a key determinant for yeast longevity. Four different yeast strains were cultured in various media, which showed similar response surface trends in biomass production and viability at day two but greatly different trends in lifespan. The pH of aging media was dependent on glucose concentration and had no apparent correlation with lifespan under conditions where amino acids and YNB were varied widely, and simply buffering the pH of media could extend lifespan significantly. Furthermore, the results showed that strain *sch9*Δ was more responsive in nutrient-sensing than the other three strains, suggesting that Sch9 (serine-threonine kinase pathway) was a major nutrient-sensing factor that regulates cell growth, cell size, metabolism, stress resistance and longevity. Overall, our findings support the notion that nutrient composition might be a more effective way than simple dietary restriction to optimize lifespan and biomass production from yeast to other organisms.

## Introduction

Calorie restriction (CR), the mere reduction of calorie intake without malnutrition [1], has become a gold standard method in aging studies, because CR was found to extend the average and maximum lifespan from yeast to primates and delay the onset of many aging-associated pathologies [2], [3]. The term CR is debated and it has been suggested that dietary restriction (DR) is a more appropriate term. Therefore, we use DR in referring to the restricted glucose level [4]. The traditional opinion on DR has been challenged in four model organisms, namely yeast [5], [6], worms [7], flies [8], [9], [10], [11], and mice [12], [13], because studies in these organisms have shown that changing the dietary components can increase their lifespans. In addition, different DR regimes extend lifespan via distinct genetic pathways [7], which suggests that nutrient balance, in addition to dietary reduction, also plays a pivotal role in regulation of longevity [14]. Although studies of yeast aging have had a significant impact on aging-related research, the new findings that nutrient composition can alter lifespan have not been systematically explored in a yeast model.

In yeast aging studies, glucose plays an important role in yeast lifespan. DR can be accomplished by only reducing the glucose concentration of growth media to extend chronological and replicative lifespan significantly, the glucose level in standard synthetic defined (SD) media could be from 2% (normal condition) to 0.5% (moderate DR) or 0.05% (severe DR) [15]. Recently, acidification of culture media was proposed to accelerate chronological and replicative aging in yeast [16], [17], [18]. Thus, it is suggested that lifespan extension by reducing the glucose level of the culture media from 2% to 0.5% or 0.05% is likely due to decreased production of organic acids and reduced media acidification [16].

Recent evidence has demonstrated that dietary amino acid compositions modulate lifespan of laboratory model organisms such as yeast [19], flies [9], and mice [13]. A report showed that reducing the amino acid concentration in the media could promote an increase in the mean and maximum replicate lifespan (RLS) of yeast [5]. Another study showed that removing preferred amino acids such as asparagine or glutamate while keeping the total amino acid concentration constant could significantly increase chronological lifespan (CLS) of yeast [6]. It is postulated that amino acids and glucose balance extends yeast lifespan and that individually reducing amino acids or glucose is a major factor in regulation of yeast longevity. It is expected that other nutrients such as minerals and vitamins may also be important in regulating yeast CLS. It would be important to know how critical these nutrients are to extending or reducing lifespan. In most aging studies, single-factorial design was employed in experiments with nutrients as the variant, resulting in an exclusive elucidation of effects of other nutrients on lifespan. By using multifactorial design, a few studies on flies have found that the nutrient balance, not DR, extends lifespan [8], [10], [20]. Multifactorial design in relation to nutrient change has not been applied in yeast aging studies to the best of our knowledge.

To uncover the relationship between nutrients and CLS, we chose a three-factor (glucose, yeast nitrogen base (YNB), amino acids)/three-level experimental design with 15 media using the SAS program (version 9.2) to arrange experiments on yeast CLS measurement (totally 240 treatments, 15 media ×16 repeats). The design was based on a classical response surface methodology (RSM) by Box–Behnken design to explore the relationships between nutrients and lifespan as well as biomass production in a wild type yeast strain [21]. We applied this design in three single gene deletion mutants (*sch9*Δ, *tor1*Δ and *sir2*Δ) to determine their changes of lifespan and biomass in response to the different nutrient compositions. Reported herein is our discovery.

## Results

### DR regime is dependent on nutrients in media

**Figure 1:**
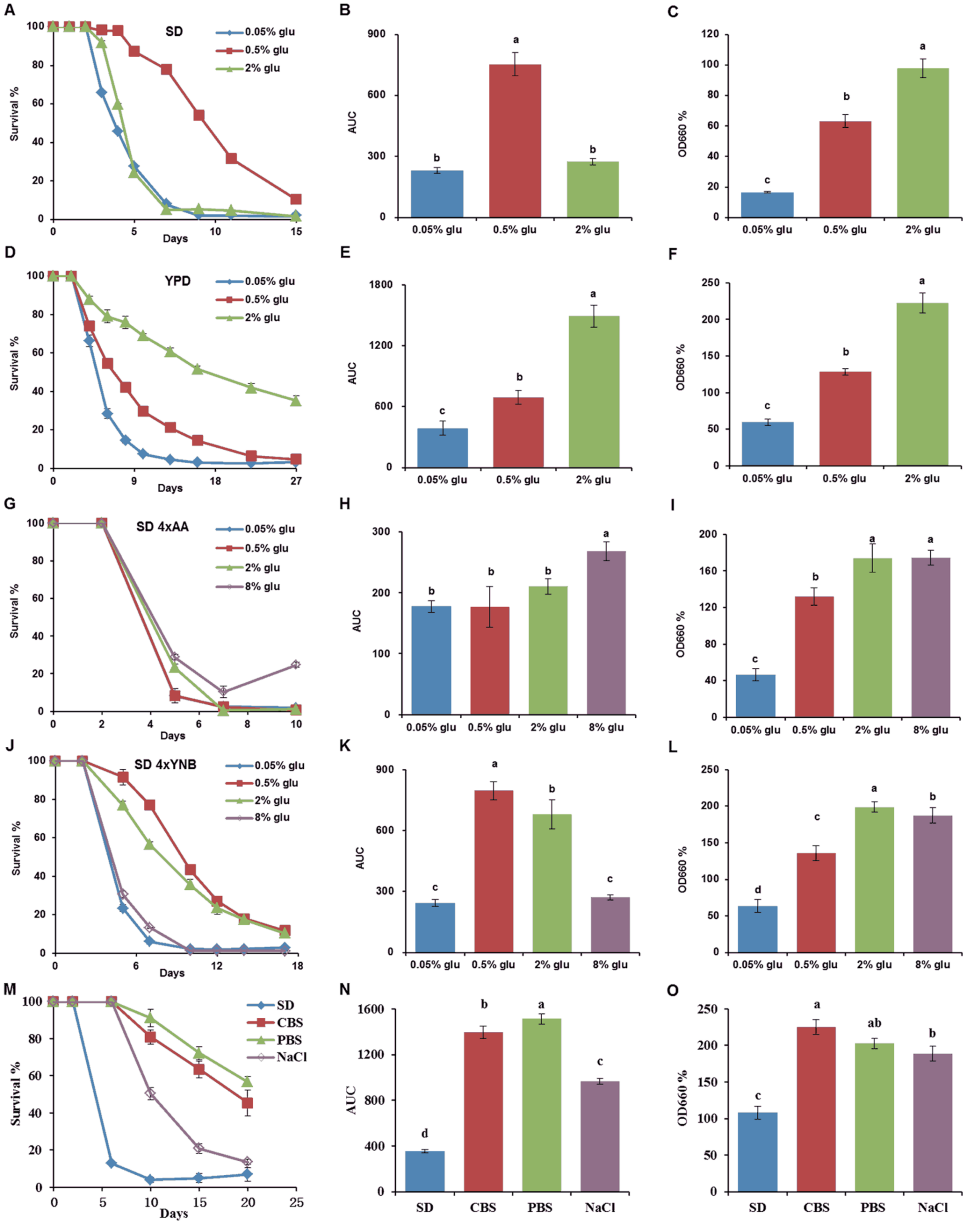
DR regime is dependent on nutrients in media. (A, B and C) Influence of glucose levels in synthetic defined (SD) media on yeast CLS. Survival curve (A) of WT strain BY4742 was inoculated in The SD medium containing 2%, 0.5% and 0.05% glucose for 15 days (mean + SEM, n = 8). (B) Area under the survival curve (AUC) represents the survival integral. DR (0.5% glu) greatly extended yeast lifespan, but further DR (0.05%) did not extend lifespan due to glucose deficiency. (C) Effect of glucose concentration in The SD medium on yeast biomass production. Yeast grown in higher glucose media produced higher biomass, and DR optimized CLS but not biomass. (D, E and F) Influence of glucose levels in the YPD medium on yeast CLS. (D) Survival curve of yeast was cultured in YPD (1% yeast extract and 2% peptone) media with different glucose levels (mean + SEM, n = 8). (E) Moderate DR (0.5%) and severe DR (0.05%) did not extend lifespan compared to normal condition (2% glu). (F) DR did not optimize CLS and biomass. (G, H and I) Influence of glucose concentrations in The SD medium containing four fold amino acids on yeast CLS. (G) Survival curve (mean + SEM, n = 8) and AUC comparison (H) shown that different glucose levels had little effect on lifespan. (I) Yeast cultured in normal condition produced higher biomass than DR condition, but further addition of glucose did not increase biomass. (J, K and L) Influence of glucose levels in The SD medium with 4-fold YNB (6.8 g/L) on yeast CLS. (J and K) DR extend lifespan, but the difference was less than that in The SD medium as show in A and B, which, due to addition of YNB, increased lifespan in 2% glucose (mean + SEM, n = 8). (L) The biomass results were similar with the observation in The SD medium with four fold amino acids. Yeast grown in 8% glucose media did not produce higher biomass than that in 2% glucose media. (M, N and O) High osmolarity and buffered media extend CLS. Yeast were inoculated into SD, The SD medium supplemented with 0.3 M NaCl (high osmolarity), and The SD medium prepared with phosphate buffer solution at (PBS, Na_2_HPO_4_ and NaH_2_PO_4_, pH 6.0) or citrate phosphate buffer solution (CBS, Na_2_HPO_4_ and citric acid, pH 6.0). (M) Survival curve (mean + SEM, n = 6) and AUC comparison (N) shown that the three media significantly prolonged CLS, as well produced higher biomass than The SD medium (O). Biomass of each aging vial at one age-point was measured as the average reading of OD values at 660 nm from 10 to 30 min in outgrowth curve. The OD value of The SD medium at day 2 was defined to be 100 relative OD value. The variance of AUC (mean + SEM, n = 8) and relative OD600 values (mean + SD, n = 8) between the treatments was compared using the Duncan’s multiple range test at *P*<0.05, different letters (a–d) showing significant differences.

Recent studies on flies suggested that the traditional observation on DR-induced longevity was mainly due to nutrient balance [8], [14], [20]. This indicates that the imbalance between nutrients resulted in lifespan reduction. To test this new insight in a yeast CLS model, we chose four media with different glucose levels to examine CLS in a commonly used wild-type yeast strain BY4742. As shown in **Figure** **1**, the four media were standard SD (synthetic defined), YPD (1% yeast extract/2% peptone/2% dextrose), SD with four-fold excess of amino acids and SD with four-fold excess of YNB (**Table** S**1**). Consistent with our previous report [22], we found that DR (0.5% glucose) increased CLS and further DR (0.05% glucose) reduced CLS, which might be due to glucose deficiency (**Figure** **1A, 1B**). Moreover, DR optimized CLS but not biomass as shown in **Figure** **1C**, and the biomass production in the media containing 2% glucose was higher than that with 0.5% glucose. This result is similar to those observed in most higher eukaryotes in that starvation does not extend lifespan, and high food intake results in high reproduction and shorter lifespan [**23**]. The difference between our results and those observations that severe DR (0.05%) extended lifespan could arise from different culture conditions [24], [25], [26].

The YPD medium was also chosen for DR studies in yeast chronological aging model [27], [28]. To our surprise, we found that the yeast cultured in the YPD medium containing 0.5% glucose had a shorter lifespan than that grown in YPD with 2% glucose, and those grown in YPD with 0.05% glucose had lifespan further reduced, similar to the results in The SD medium (**Figure** **1A, 1D, 1E**). For biomass, the observation was also similar to the results in The SD medium that higher glucose concentrations produced higher biomass; however, the biomass at the same glucose level was significantly higher than that of The SD medium (**Figure** **1C, 1F**). The YPD medium was rich in nutrients, we propose that 2% glucose in the YPD medium could mimic DR condition of the 0.5% glucose in The SD medium to induce longevity of yeast, and that lifespan reduction in 0.5% and 0.05% glucose YPD would be due to glucose deficiency.

For the preliminary study of amino acids and YNB in our yeast aging model, four-fold of total amino acids were added into the The SD medium containing different glucose levels. As shown in **Figure** **1G-I**, the addition of total amino acids did not extend CLS, but increased biomass production, and the DR condition (0.5% glucose) did not produce a longer lifespan than the normal condition (2% glucose). Interestingly, our results showed that the addition of four-fold YNB (6.8 g/L) significantly extended CLS and increased biomass production than that of the The SD medium with 2% glucose (**Figure** **1J–L**). Yeasts in the high YNB media with 0.5% glucose had longer CLS than that with 2% glucose. Therefore, the results from high amino acids and YNB media suggested that the concentrations of both components played important roles in regulation of yeast CLS. Furthermore, it was observed that higher glucose levels produced more biomass from 0.05% to 2% glucose, but resulted in greatly different CLS. In contrast, 8% glucose could not further increase biomass in both high amino acids and YNB media. This may be due to shortages of YNB and amino acids relative to the high glucose content (**Figure** **1I, 1L**).

Previous studies propose that acetic acid induced cell death is the key mechanism of chronological aging in yeast in standard media, and environmental and genetic interventions via increasing cellular resistance to acetic acid can extend CLS [16], [17], [29]. In this study, our results are consistent with these reports, and show a high osmolarity media (0.3 M NaCl) and buffered media (pH 6.0) can significantly extend the CLS of yeast (**Figure** **1M, 1N**). Interestingly, the high osmolarity and buffered media produced more biomass than the standard The SD medium even though they had the same amounts of glucose and other nutrients (**Figure 1O**), and the buffered media induced higher biomass was also observed in a previous study [**16**].

### Development of statistical design of experiments for evaluation of nutrition, biomass and lifespan

To determine the relationships between nutrients and lifespan as well as biomass production of yeast, a 15-media experiment with three-factors (glucose, YNB, amino acids)/three-levels (–1, 0, 1) was selected according to the classical RSM Box–Behnken design (**Table** **1**). This design can explore the relationships between several explanatory variables (nutrients) and response variables (lifespan, biomass) under the design region by using a minimal number of experimental runs. The concentrations of three factors, i.e. glucose (0.5 to 5.5%), amino acids (0.5 to 3.5 ×) and YNB (0.85 to 9.35 g/L), were chosen to test conditions known to alter lifespan in a typical The SD medium (**Table S1**). The wild-type strain BY4742 and three single gene deletion strains (*sch9*Δ, *tor1*Δ, *sir2*Δ) were chosen because the three genes are highly conserved from yeast to mammals as well as play critical functions during aging regulation in a range of model organisms [23], [30], [31], [32]. Thus, this experiment contained a total of 1024 runs, including the standard The SD medium (4 strains ×16 media ×16 repetitions).

**Table 1:** The three-factor/three-level response surface methodology of Box–Behnken design and pH, biomass and lifespan values of different cultures in the wild-type and *sch9*Δ strains.

Run	Factors & levels (coded)						pH					Biomass (OD660%)		Lifespan (AUC)	
	AAs (1×)		YNB (g/L)		Glucose (%)		Day 0	WT day 2	WT day 4	*sch9*Δ day 2	*sch9*Δ day 4	WT	*sch9*Δ	WT	*sch9*Δ
1	0.5	(–1)	0.85	(–1)	3.0	(0)	4.73	3.00	3.09	3.00	3.12	11.7	36.5	594.0	413.8
2	0.5	(–1)	9.35	(1)	3.0	(0)	4.60	3.21	3.27	3.26	3.34	52.1	59.7	559.7	303.9
3	3.5	(1)	0.85	(–1)	3.0	(0)	4.75	3.65	3.60	3.88	4.04	50.9	47.6	344.4	2455.8
4	3.5	(1)	9.35	(1)	3.0	(0)	4.72	4.80	4.42	4.67	4.24	226.8	263.1	762.0	678.1
5	2.0	(0)	0.85	(–1)	0.5	(–1)	4.77	5.46	6.61	4.90	6.55	29.2	48.6	408.6	285.6
6	2.0	(0)	0.85	(–1)	5.5	(1)	4.72	3.17	3.36	3.25	3.37	45.2	55.9	898.2	2701.4
7	2.0	(0)	9.35	(1)	0.5	(–1)	4.71	5.88	5.93	5.88	5.93	53.1	69.5	688.9	770.2
8	2.0	(0)	9.35	(1)	5.5	(1)	4.71	3.54	3.56	3.67	3.49	105.9	191.3	443.5	780.0
9	0.5	(–1)	5.10	(0)	0.5	(–1)	4.71	3.82	3.94	3.63	3.78	30.9	45.3	894.6	318.2
10	3.5	(1)	5.10	(0)	0.5	(–1)	4.76	6.67	6.83	6.25	6.66	40.8	65.7	426.4	319.9
11	0.5	(–1)	5.10	(0)	5.5	(1)	4.68	2.73	2.81	2.79	2.82	55.6	57.0	308.6	504.1
12	3.5	(1)	5.10	(0)	5.5	(1)	4.73	3.89	3.84	4.16	4.58	245.3	284.9	831.0	2397.2
13	2.0	(0)	5.10	(0)	3.0	(0)	4.73	4.11	3.55	4.11	3.33	112.2	171.0	760.1	497.4
14	2.0	(0)	5.10	(0)	3.0	(0)	4.73	4.10	3.57	4.13	3.35	126.5	185.0	778.0	496.3
15	2.0	(0)	5.10	(0)	3.0	(0)	4.73	4.11	3.53	4.14	3.36	121.8	176.8	775.1	502.3
SD	1.0		1.7		2.0		4.72	3.45	3.52			108.4		356.4	
CBS	1.0		1.7		2.0		6.00	5.92	5.89			225.1		1395.6	

The pH, biomass and lifespan data are presented as mean pH of several biological replicates.

### Nutrient composition is a key factor for longevity of yeast

**Figure 2:**
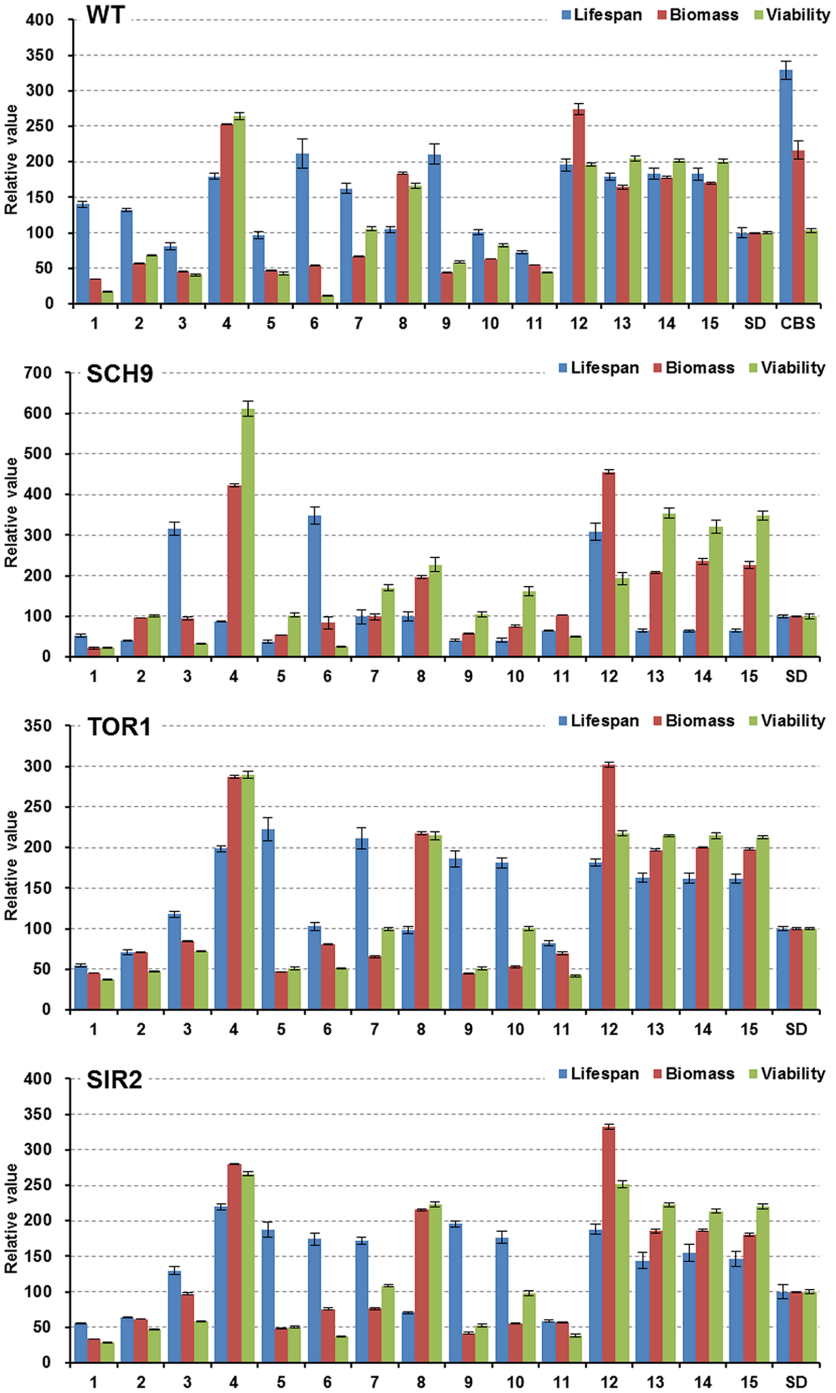
Comparison of relative lifespan, biomass and viability in the four yeast strains (WT, *sch9*Δ, *tor1*Δ, and *sir2*Δ). AUC represents the survival integral for lifespan comparison, the AUC of yeast aging in The SD medium is defined as 100%, and error bars represent SEM within 16 replicates. Biomass production was measured as the average values at OD660 of each media from day 10 to day 22 (see **Figure** **S2**), the biomass of The SD medium was defined as 100%, (mean + SEM, n = 16). Viability was the survival at day 2, and the survival of SD at day 2 was defined as 100%, (mean + SEM, n = 16).

**Figure 3:**
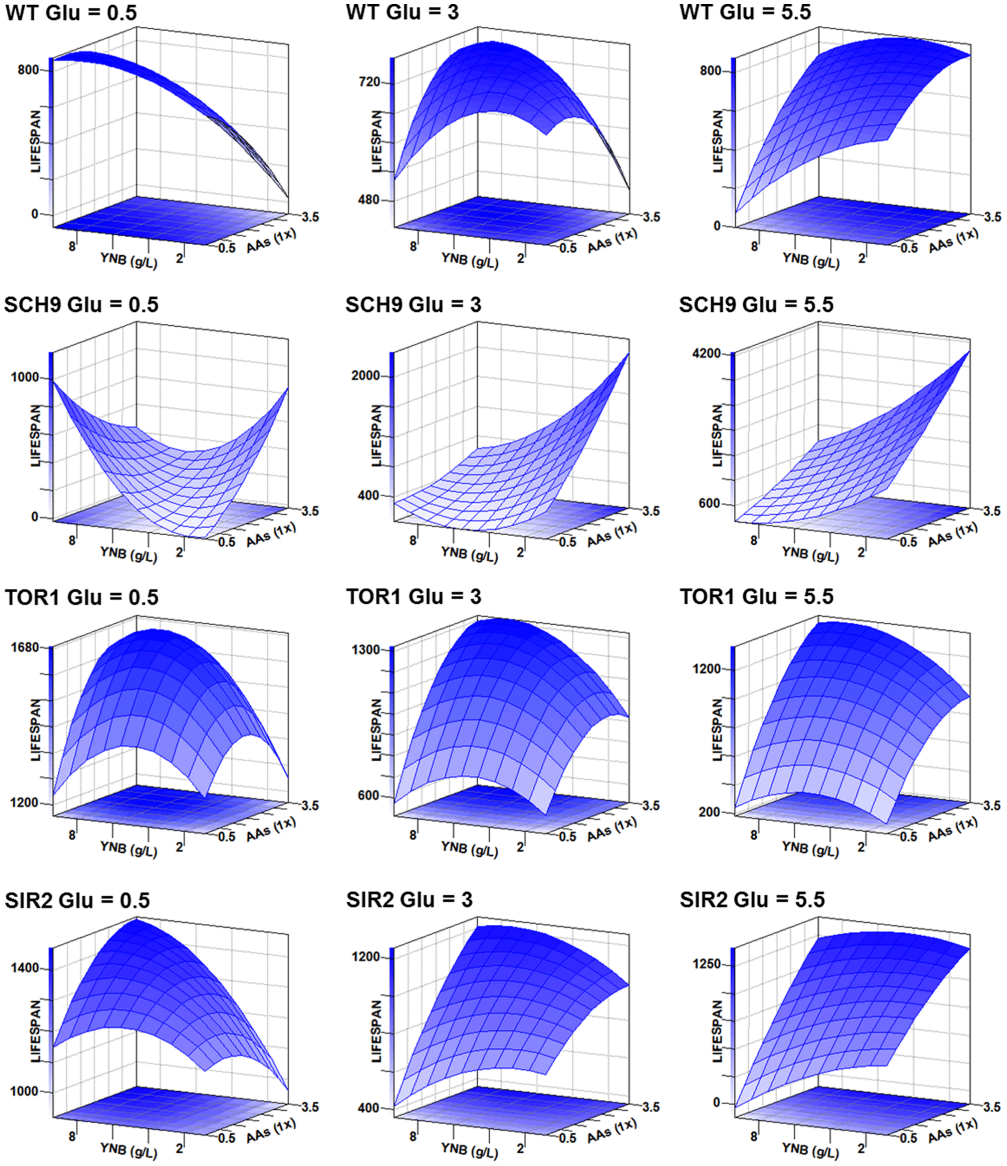
Response surfaces for lifespan of the four yeast strains (WT, *sch9*Δ, *tor1*Δ, and *sir2*Δ) cultured at various concentrations of amino acids (AAs), glucose (Glu), and yeast nitrogen base (YNB). For each strain, three AA/YNB-lifespan plots are shown at specific glucose levels 0.5% (low), 3% (media) and 5.5% (high) respectively. The AAs concentration ranged from 0.5× to 3.5× and YNB from 0.85 to 9.35 g/L. The surface-response in *sch9*Δ was clearly different from that in WT, *tor1*Δ and *sir2*Δ, while these plots displayed similar trends in *sir2*Δ and *tor1*Δ. All these surface-response plots were generated automatically by SAS program, and those surface plots for biomass production are seen in **Figure 4**.

To elucidate whether nutrient balance is an important factor for longevity of yeast, we did an analysis from the following aspects:

Firstly, the relative lifespan of the four strains (WT, *sch9*Δ, *tor1*Δ, and *sir2*Δ) is shown in **Figure** **2**, and the response surfaces for the lifespan of the four strains cultured at various concentrations of amino acids, glucose, and YNB are plotted in **Figure** **3**. For the WT, only the middle glucose level (3%) had an optimal lifespan within the testing concentration ranges of amino acids and YNB. However, the response surface plots of *sch9*Δ were greatly different from those of the WT at the three glucose levels. For the *tor1*Δ and *sir2*Δ, the trends of response surfaces bore some similarities, and this was consistent with the result that *tor1*Δ and *sir2*Δ had a good correlation in lifespan change (*vide infra*). Overall, these results clearly showed that yeasts cultured in media containing different ratios of three types of nutrients had diverse lifespans. The diverse change of lifespan in these media among the four strains tested suggested that nutrient composition, instead of glucose alone, played a more important role in regulation of lifespan.

Secondly, among the 15 media, the lifespan of WT and *sch9*Δ, respectively, had little correlation with that of the other strains, only *tor1*Δ and *sir2*Δ had a good correlation (r = 0.87, *P*<0.001) in lifespan change (**Table** **2**). This means that strains WT, *sch9*Δ and *tor1*Δ/*sir2*Δ cultured in the same media had different lifespans (**Figure** **2**). It is noticeable that, in some cases, *sch9*Δ, *tor1*Δ, and *sir2*Δ strains had shorter lifespans than the WT (**Figure** **S1**). Previous findings show that single deletion of any one of the three genes changed the lifespan (increased or reduced). Our results suggested that, on top of the DR regime, the changes in lifespan may also be dependent on the nutrient compositions of the media used in the experiment [14], [33].

**Table 2:** Correlation among lifespan, biomass and viability at day 2 for the 15 media in the four *Saccharomyces cerevisiae* strains.

			WT			*sch9*Δ			*tor1*Δ			*sir2*Δ	
		Lifespan	Biomass	Viability	Lifespan	Biomass	Viability	Lifespan	Biomass	Viability	Lifespan	Biomass	Viability
	Lifespan		0.40	0.38	0.18	0.40	0.37	0.30	0.39	0.37	0.52	0.37	0.37
WT	Biomass	0.14		0.94	0.13	0.97	0.79	0.31	0.99	0.95	0.31	0.99	0.96
	Viability	0.16	<.0001		–0.11	0.88	0.92	0.42	0.92	0.98	0.34	0.89	0.98
	Lifespan	0.52	0.64	0.71		0.24	–0.26	–0.12	0.20	0.00	0.20	0.28	–0.01
*sch9*Δ	Biomass	0.14	<.0001	<.0001	0.39		0.75	0.33	0.96	0.87	0.37	0.97	0.88
	Viability	0.17	0.00	<.0001	0.35	0.00		0.47	0.77	0.90	0.42	0.72	0.86
	Lifespan	0.28	0.26	0.12	0.67	0.23	0.08		0.23	0.37	0.87	0.27	0.37
*tor1*Δ	Biomass	0.15	<.0001	<.0001	0.47	<.0001	0.00	0.40		0.94	0.26	0.99	0.95
	Viability	0.18	<.0001	<.0001	0.99	<.0001	<.0001	0.18	<.0001		0.33	0.92	0.99
	Lifespan	0.04	0.27	0.22	0.48	0.17	0.12	<.0001	0.35	0.23		0.30	0.31
*sir2*Δ	Biomass	0.17	<.0001	<.0001	0.32	<.0001	0.00	0.32	<.0001	<.0001	0.28		0.93
	Viability	0.17	<.0001	<.0001	0.98	<.0001	<.0001	0.17	<.0001	<.0001	0.27	<.0001	

Pearson coefficient (r) is shown in top right of the table and corresponding *P*-value is shown in bottom left of the table.

Thirdly, the linear and the quadratic parameter estimates for the lifespan are presented in **Table** **S2**. The results showed that although not all terms had significant effects on the lifespan, the estimates of the same term had marked differences among the four strains, which was different from the biomass results (*vide infra*). It further suggests that nutrient composition is an important factor for longevity of budding yeast and the three nutrients and their interactions play different roles in the lifespan of different strains.

Lastly, a few media induced regrowth of yeast cells, especially media 6 (2× AAs, 0.85 g/L YNB and 5.5% glucose) (**Figure** **S1**). The regrowth is important for adaptation to starvation conditions, and many laboratory wild-type microorganisms have adaptive regrowth when usually 90–99% of the population dies. Longo *et al.* suggested that adaptive regrowth is correlated with increased mutation frequency, which allows mutants to re-enter the cell cycle under starvation conditions [29], [34]. Thus, they proposed to monitor chronological aging of yeast in water that can prevent any occurrence of adaptive regrowth, which might confound the explanation of survival data [35]. However, the observation in media 6 might be caused by an imbalance of the low YNB content. In addition, this media as well as a few others that produced longer lifespans in all four strains suggested that nutrient composition a key determinant for yeast longevity.

### Biomass production of the four strains has similar changes in response to nutrient composition

During chronological aging, the total cell number in the media (defined as biomass production) had little change (**Figure** **S2**), but the number of living cells (survival %) had been reducing stage by stage. As listed in **Table** **2**, the biomass and viability had a good positive correlation among the four strains, which suggests that viability at day 2 may represent the biomass production of the media, and there was no need to measure the OD660 values of the media at each aging point as the biomass.

**Figure 4:**
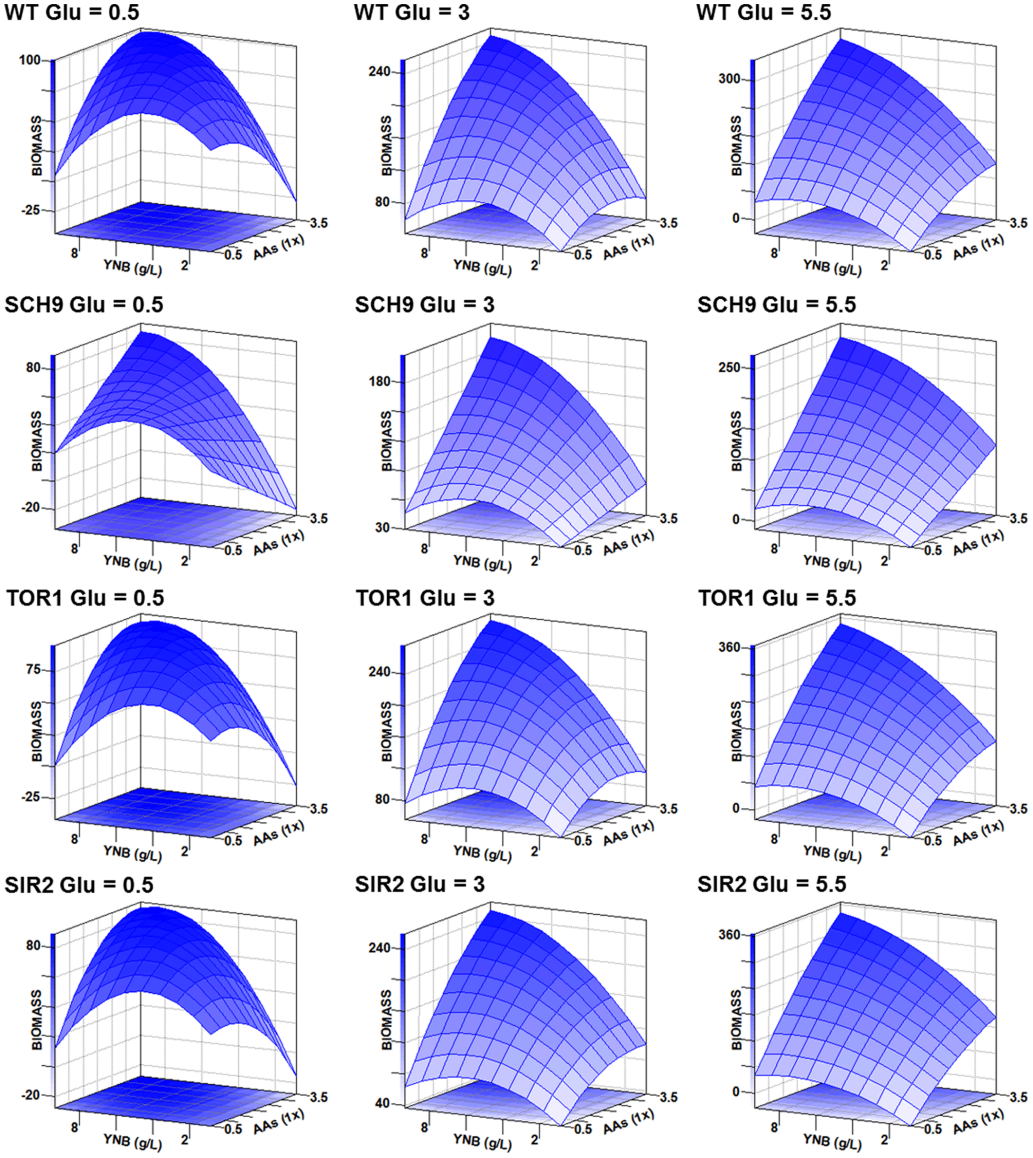
Response surfaces for biomass production of yeast cultured at various concentrations of amino acids (AAs), glucose (Glu), and yeast nitrogen base (YNB). For each strain, three AA/YNB-lifespan plots are shown at specific glucose levels 0.5% (low), 3% (media) and 5.5% (high) respectively. The AAs concentration ranged from 0.5× to 3.5× and YNB from 0.85 to 9.35 g/L. These surface-response plots displayed similar trends in the four strains. All these surface-response plots were generated automatically by SAS program, and those surface plots for lifespan are seen in **Figure 3**.

It is known that different strains had significant lifespan changes in various media; however, this result was not observed in biomass production. On the contrary, the biomass production of the four strains had similar change trends among the 15 media. Firstly, the Pearson coefficient (r) of WT with SCH9, TOR1, and SIR2 were 0.97 (*P*<0.0001), 0.99 (*P*<0.0001), and 0.99 (*P*<0.0001), respectively (**Table** **2**), but for lifespan, the corresponding r values were 0.18 (*P* = 0.52), 0.30 (*P* = 0.28) and 0.52 (*P* = 0.04). Secondly, all linear terms (AA, YNB and GLU) and all cross terms (AA*YNB, AA*GLU and YNB*GLU) had positive effects, and all quadratic terms (AA*AA, YNB*YNB, GLU*GLU) had negative effects on biomass production in the four strains and most estimates of these terms were significant (*P*<0.005). However, these results were clearly different from the data on lifespan (**Table** **S2**). Thirdly, the response surface plots illustrated that biomass production of the four strains cultured in various concentrations of amino acids, glucose and YNB had similar trends (**Figure** **4**), but the lifespan plot among the four strains showed diverse trends (**Figure** **3**).

For the optimal media for maximizing biomass production, as can be seen in **Figure** **4**, these response surface plots indicate that only the low level of glucose (0.5%) media had a maximal biomass from the coded concentration range of amino acids (0.5–3.5 ×) and YNB (0.85–9.35 g/L), while the media with middle or high levels of glucose had not, and it meant higher nutrient amounts in media produced higher biomass for all the four stains.

### SCH9 is more responsive in nutrient-sensing than the other three strains

To explore the responses of the four strains to various nutrient compositions, we studied cell growth in the 15 media, SD and the YPD medium. As shown in **Figure** **S3**, the yeast cells of WT, *tor1*Δ, and *sir2*Δ grew well, and biomass production was dependent on the available nutrients in the media. In contrast, growth of *sch9*Δ cell was greatly disturbed in some media, the cells cultured in media 1, 3, 5, 6, and SD did not grow better than the yeast in other media. Nevertheless, we found that *sch9*Δ grew well and the growth curves were similar to those of the other strains when cultured in a few media such as 7, 11 and YPD. Thus, this result could indicate that the growth of *sch9*Δ was more sensitive to nutrients than the other three strains.

We also observed that the phenotype of strain *sch9*Δ cells would gather together in several media (**Figure** **S4**). Moreover, these aggregative cells formed macroscopic agglomerates and settled to the bottom of sample vials. In addition, this aggregation phenomenon can be seen from the growth curve (**Figure** **S3**) that had a clear descending trend after the maximal OD660 value, such as 1, 2, 3, 6, 8, 12, 13, 14, 15 and SD. However, this phenomenon was not found in other strains (**Figure** **S3**, **S4**), and the aggregation did not relate to biomass production and lifespan (**Figure** **2**). Overall, these results suggest that Sch9 regulates cell growth was highly sensitive to the nutrient ratios.

### Aging media pH is dependent on glucose concentration and has no apparent correlation with lifespan

In the yeast CLS model, acidification of the culture media accelerates chronological aging when cells are cultured in a SD liquid media [16], [17], [18]. The pH of a yeast aging culture is dependent on the media composition, especially glucose concentration. Yeast cells metabolize glucose and other substrates such as amino acids to produce some acids via glycolysis and citric acid cycle such as acetic, pyruvic and succinic acids that acidify the media. In this study, we examined pH changes in different media in wild-type and *sch9*Δ (**Table 1**) and analysed the correlations among pH, amino acid, YNB, glucose, lifespan and biomass (**Table 3**). We found that the pH of aging cultures ranged from 2.7 to 6.8 and pH at day 2 and day 4 had no significant difference, which was consistent with a previous study [16]. In addition, the pH of wild-type cultures had a similar trend instead with that of *sch9*Δ strain, which might indicate that pH changes of different media were independent of deletion of *SCH9*. Furthermore, the pH of the two strains at two age-points had a strong correlation with the glucose content in the media, but had no correlation with lifespan or biomass. This result suggested that nutrient composition could offset the effect of extracellular low pH to influence yeast CLS under the current experimental conditions, possibly due to intracellular buffering capacity [36]. The pH as one determinant of CLS was also observed only in a few cases in this study (media 7 versus media 8). It should be noted that simply buffering the pH of the standard The SD medium (CBS) could result in CLS that is greater than any of the 15 media compositions (**Table 1**) in WT strain. For unbuffered conditions, previous studies focused on glucose percentage in media and did not consider amino acids and YNB composition, thus it was concluded that low pH accelerates chronological aging and pH neutralization increases CLS in the standard The SD medium [17], [18]. In this study, we compared the lifespan of yeast in various media with changes in YNB and amino acids, which likely affected the chronological viability by alleviating the negative impact of media acidification and acetic acid toxicity.

**Table 3:** Correlations among amino acid, YNB, glucose, pH, lifespan and biomass for the 15 media in the wild-type and *sch9*Δ strains.

		nutrient			WT				*sch9*Δ			
		AA	YNB	Glucose	pH day 2	pH day 4	Lifespan	Biomass	pH day 2	pH day 4	Lifespan	Biomass
	AA		0.00	0.00	0.53	0.42	0.00	0.54	0.60	0.50	0.48	0.57
nutrient	YNB	1.00		0.00	0.18	0.04	0.10	0.46	0.24	–0.01	–0.37	0.41
	Glucose	1.00	1.00		–0.72	–0.74	0.03	0.42	–0.65	–0.67	0.53	0.41
	pH day 2	0.06	0.55	0.01		0.96	–0.07	0.04	0.99	0.93	–0.27	0.03
WT	pH day 4	0.15	0.90	0.0056	<.0001		–0.16	–0.11	0.92	0.98	–0.28	–0.12
	Lifespan	0.99	0.74	0.92	0.83	0.60		0.35	–0.04	–0.17	0.25	0.36
	Biomass	0.06	0.12	0.16	0.90	0.73	0.25		0.12	–0.06	0.20	0.97
	pH day 2	0.03	0.44	0.02	<.0001	<.0001	0.90	0.70		0.91	–0.17	0.11
*sch9*Δ	pH day 4	0.08	0.98	0.01	<.0001	<.0001	0.59	0.85	<.0001		–0.14	–0.05
	Lifespan	0.09	0.21	0.06	0.37	0.35	0.41	0.51	0.57	0.64		0.29
	Biomass	0.04	0.16	0.16	0.93	0.70	0.22	<.0001	0.73	0.88	0.33	

Pearson coefficient (r) is shown in top right of the table and corresponding *P*-value is shown in bottom left of the table.

### The optimal The SD medium for yeast

Based on the four strains used in this study, the media 12 would be the best one for yeast since it produced the maximal biomass and lifespan (**Table** **S3**). However, it produced a shorter lifespan than the YPD and pH buffered The SD medium in the WT strain (**Figure 2**, **Table 1**). It has a slight influence on cell growth in *sch9*Δ (**Figure** **S3**, **S4**). In addition, our results showed the WT cultured in YPD (2% glucose) had a remarkable longevity (**Figure** **1**). The other three strains (data not shown) also showed lifespan extension. All four strains grew well in YPD and there was no aggregation in *sch9*Δ (**Figure** **S3**). However, not all the chemicals used in YPD are known, thus YPD was not an ideal media for investigating the effects of nutrient components on aging.

On the other hand, the standard The SD medium is better suited for yeast aging study, and we can conveniently modify amino acid compositions to identify optimal amino acid requirements of specific mutants. For yeast aging study, development of an ideal The SD medium mimicking YPD that can meet cell growth requirements and achieve longevity for most strains would be interesting but hard to achieve. In this study, we found that not only glucose and amino acids but also YNB played a significant role in regulating lifespan and biomass production of yeast strains, especially of *sch9*Δ. Thus, modification of amino acids and YNB composition was important for development of a better The SD medium for yeast aging study.

## Discussion

In this study, we used a high throughput screening assay to comprehensively evaluate the relationships between nutrients (glucose, amino acids and YNB) and lifespan as well as biomass production in four yeast strains (wild-type BY4742, *sch9*Δ, *tor1*Δ, and *sir2*Δ). Experimental design based on the classical RSM with a total of 1024 treatments (4 strains ×16 media ×16 repeats) was applied to show that different strains cultured in various media had similar response surface trends in biomass production and viability at day 2, but very different trends in lifespan. All the three groups of nutrients and their interactions played different roles in regulation of lifespan of different strains. In addition, we propose that viability at day 2 might represent the biomass production of the media since it had a good correlation with measured biomass based on the OD660 values of the media. Furthermore, our findings indicate that lifespan extension by DR regime was dependent on nutrients in media and that nutrient composition was a key factor for longevity of yeast.

Recent studies have revealed that nutrition influences the biological aging process in different model organisms, especially the macronutrients including carbohydrates, fats, proteins, and water [8], [10], [11], [37]. This information is particularly important because of its potential for developing interventions to prevent age-related diseases and promote healthy aging.

The common media for yeast aging study is the SD medium containing a limited amount of nutrients, which mimics yeast survival in the wild [35]. The The SD medium contains glucose, YNB, ammonium sulphate, and an amino acid mixture. YNB contains salts, vitamins and trace elements (**Table** **S1**). For the standard The SD medium, it has been shown that DR (0.5% glucose) can extend yeast replicative and chronological lifespan in various strains, as compared to the normal condition (2% glucose). However, recent studies on flies also suggest that the traditional observation on DR-induced longevity was mainly due to nutrient balance. This indicates that the imbalance between dietary and other nutrients resulted in lifespan reduction under normal conditions [8], [14], [20]. In addition, DR in mammals is commonly defined as reduction in dietary intake without malnutrition by 10% to 50% of *ad libitum* [2], [23]. However, DR in yeast is modeled by glucose restriction and reducing the glucose level in the The SD medium from 2% (normal condition) to 0.5% (moderate DR) or to 0.05% (severe DR) can extend lifespan of different yeast stains [15].

Recent studies suggest that the low glucose induced longevity was partially due to decreased production of acetic acid and reduced media acidification for two possible reasons: (1) acetic acid was identified as an extracellular mediator of cell death during chronological aging, and it was demonstrated that environmental interventions by reducing or eliminating acetic acid increased CLS, such as via DR, using non-fermentable carbon source, or transferring cells to water [16]; (2) pH neutralization was demonstrated to protect against reduction in RLS and CLS in yeast [17], [18], [29], extracellular acidification of the culture media could cause intracellular damage that subsequently limited the cell replicative potential, and the reduced RLS and CLS could be extended by buffering the pH of media to 6.0 [18].

In this study, we also examined impact of DR, growth on a non-fermentable carbon source, transferring yeast to water (unpublished data), deletion of *SCH9* and *RAS2*, and growth in high osmolarity or buffered media. Consistent with previous observations, we found that all these factors could extend yeast CLS. However, our results also showed that low glucose level media (low acetic acid content and high pH) resulted in CLS reduction (**Fig. 1** **,** **2** **,** **3**), and that the relatively high glucose media (high acetic acid content and low pH) extended CLS (media 6 versus media 5 or media 12 versus media 10). In addition, we measured the acetic acid contents of different cultures of wild-type and *sch9*Δ at day 2 and found the acetic acid concentration was relatively low (<10 mM) and could only be detected in a few high glucose media (unpublished data). This indicates that acetic acid might not always be the key determinant of CLS, which is supported by others [38]. It is likely that the effect of acetic acid on yeast survival is condition-dependent, such as media composition, nutrient composition, and buffering capacity of both extracellular and intracellular media [36].

Due to the complexity of the factors impacting yeast ageing, at present, it is still not clear why our observations differ from prior studies (low glucose, <0.5%). One possibility might be that our study focused on the effects of nutrients (not only glucose, but also YNB and amino acids) on CLS of yeast in the SD medium. YNB and the initial amino acid composition could alter the intracellular pH of the aging culture, affect the yeast cell survival, and negate acetic acid toxicity. Another possibility might be that our conditions were not optimized for CLS extension by low glucose (< 0.5%). It should be noted that the different results were also likely attributable to differences in the media composition and culture conditions. For example, we modified the amino acid composition of the SD medium (**Table S1**), which is different from prior studies. Although we followed similar composition of some amino acids such as L-arginine, L-methionine, L-serine, L-tryptophan, and L-tyrosine [22], [39], we used a shaker incubator with a high capacity of 600 aging culture vials (4 mL, 15×45 mm, with plastic caps) with a relatively small media volume of 1 mL, which is greatly different from other laboratories, where bigger culture containers with more liquid media, were used. This could result in different cell population, culture aeration and oxidative metabolism [38]. Furthermore, yeast CLS is influenced by additional factors, including strain auxotrophy, the way the cultures are aerated, the use of 96-well microplates, the use of spectrophotometric vs. CFU-based methods for quantifying viability. All these factors may have contributed to the different observations. The current work did not necessarily disprove previous findings of acetic acid/pH as one important factor for yeast aging, although cautions should be exercised in interpretation of data (e.g. experimental conditions applied). The findings from our laboratories need to be validated by different laboratories and further work is needed to understand the reasons for these different observations about the effect of pH on lifespan, for example, using buffered media with different nutrient composition.

YPD would be a good media for cell growth and longevity study since it contains yeast extract and peptone, which are rich in many types of nutrients. It has applications in diverse yeast strains [27], [28]. The YPD medium was chosen for the DR study in yeast chronological aging model mainly due to two factors. Firstly, it allows isolation of quiescent and non-quiescent cells from the stationary phase cultures grown in the YPD medium [28], [40]. Secondly, the shape of mortality curves for yeast grown in the nutrient-rich YPD medium was similar to the mortality patterns observed in multicellular eukaryotes [41]. Furthermore, yeast grown in the YPD medium containing 0.2% or 0.5% glucose lived significantly longer than that grown at 0.05%, 1% or 2% glucose [27], which suggests that the glucose level affects lifespan of yeast as observed in many higher eukaryotes.

It has been reported that amino acid balance plays a critical role in regulation of lifespan in rat, fly and yeast, independently of DR [9], [13], [19], [42], [43], [44]. Methionine restriction can decrease visceral fat mass, preserve insulin action, and prolong lifespan in rats independent of DR [43]. In *Drosophila*, adding methionine alone to DR condition increased fecundity as much as that under regular feeding and without reducing lifespan [9]. In *Saccharomyces cerevisiae*, a few studies have shown that reduction in methionine increased the replicative lifespan (RLS) [42] and removal of either asparagine or glutamate can significantly increase CLS [6]; furthermore, addition of isoleucine, threonine, valine, and leucine can extend CLS [19]. However, these studies focused only on amino acids and did not consider other nutrients present in the media; thus, the relationship between glucose and amino acids in regulation of lifespan was still not established.

In this study, we found that not only glucose and amino acids, but also YNB, played a significant role in regulation of yeast lifespan and biomass production; the three groups of nutrients and their interactions played different roles in regulation of lifespan of different strains. Our objective was not to produce an optimal media for yeast aging studies, but to demonstrate the fact that optimizing culture media by single nutrient variation is not sufficient to maximize the lifespan or biomass of yeast, although some studies have confirmed that glucose or a few amino acids are important for longevity of yeast and simple modification of one of these nutrients can greatly extend lifespan [6], [19]. With a suitable experimental design, we can obtain an optimal The SD medium to maximize the lifespan for a specific yeast strain.

For lifespan, strain *sch9*Δ seemed to be more sensitive to nutrients, since more terms had significant (*P*<0.001) effects on lifespan in *sch9*Δ than in the other strains (**Table** **S2**). The AGC kinase Sch9 is a substrate of multiprotein complex TORC1, and its function may be similar to the mammalian TORC1 substrate S6K1 [45]. In yeast, Sch9 regulates cell growth and cell size, the absence of Sch9 activity causes a small size phenotype and distinct growth defect, while increasing lifespan by seven-fold [46], [47], [48]. The Tor-Sch9 pathway was thought previously to be a nutrient-sensing pathway [23], [49].

In this study, we have confirmed and extended previous work by showing that deletion of *TOR1* regulates yeast CLS subjected to amino acids and glucose concentrations (**Table S2**). It is remarkable that our data have shown that the *sch9*Δ strain is more responsive in nutrient-sensing than the other three strains. Sch9 protein kinase was proposed previously as a central coordinator of protein synthesis [50] in promoting ribosome biogenesis and ribosomal protein gene expression [51]. Thus, it is possible that Sch9 acts as a major nutrient-sensing factor to regulate cell growth, cell size, and stress resistance through control protein synthesis. However, further experiments on quantifying Sch9 activity in different media are warranted to delineate the role of *SCH9* plays in our system.

In conclusion, our findings indicate that lifespan extension by DR may be partially dependent on nutrient composition and could be abolished by providing yeast with different nutrient compositions. Furthermore, our results show that *sch9*Δ is more nutrient-sensitive than the other three strains tested. Modification of amino acids and YNB compositions is an important factor to consider if one were to develop an optimal The SD medium that can meet the cell growth requirements and enable longevity of most strains for yeast aging studies and evaluation of anti-aging activity of small molecules. Our results also document that nutrient composition is an important factor for yeast CLS. Different yeast strains cultured in various media exhibited similar response surface trends in biomass production, but showed greatly different trends in lifespan. The three nutrients (glucose, amino acids and YNB) and their interactions played different roles in affecting lifespans of different strains. Taken together, our findings suggest that nutrient composition is an effective way to optimize lifespan and biomass production in yeast.

## Materials and Methods

### Materials

The wild-type strain *Saccharomyces cerevisiae* BY4742 (MATα *his3*Δ1 *leu2*Δ0 *lys2*Δ0 *ura3*Δ0) and single gene deletion mutant strains in the BY4742 genetic background were obtained from Thermo Scientific Open Biosystems (Huntsville, AL, USA). The culture of each yeast reference strain was aliquoted into 10 µL and stored at –80°C. All L-amino acids were from GL Biochem (Shanghai, China), yeast nitrogen base w/o amino acids (YNB), peptone, agar, yeast extract were from Amresco (Solon, OH, USA). YPD Broth and other chemicals were from Sigma-Aldrich Chemical Company (Singapore).

### Experimental design and statistical analysis

The three experimental factors under study were glucose, amino acids, and YNB in synthetic defined (SD) media (**Table** **S1**). Values for these parameters were chosen to test conditions known to produce significant effects on lifespan from our previous study [21]. To test for the curvature of the responses, three levels of each nutritional parameter were required (**Table 1**). A Box-Behnken design based on response surface methodology (RSM) was chosen to estimate the responses of both the linear and the quadratic behavior over the design region to minimize the number of experiments [21]. This design was generated from the SAS program (version 9.2, SAS Institute Inc, Cary, NC, USA), and required a total of 15 runs, including three center points (run 13, 14 and 15).

### Lifespan, biomass and yeast cell growth assay

The determination of chronological lifespan of yeast was carried out according to the method described previously [22], [39]. In brief, the yeast cells were prepared by transferring a streaked strain from frozen stocks onto YPD (1% yeast extract/2% peptone/2% dextrose) agar plates. After incubating the cells at 30°C for 2 days or until colonies appeared, a single colony was picked and inoculated into 1.0 mL YPD liquid media (Sigma YPD Broth, Louis, MO, USA) in a 4-mL glass vial and cultured at 30°C for 2 days in a flat incubator at 200 rpm. The 2-day YPD culture was diluted with autoclaved 18 mΩ milli-Q grade water (1:10) and stored in refrigerator at 4°C for at least 24 h. After one day incubation at 4°C, 5 µL (≈ 1×10^4^ cells) of the diluted culture was transferred to 1.0 mL of different aging media and maintained at 30°C, 200 rpm for the entire experiment. After 2 days of culture in aging media, the cells reached stationary phase and the first age-point was ready to be taken. Subsequent age-points were taken every 2–4 days. For each age-point, 5.0 µL of the mixed culture was pipetted into each well of 96-well microplate (Nunc, Rochester, NY, USA). One hundred microliter YPD medium was then added to each well. The cell population was monitored with a Synergy HT microplate reader (BioTek, Winooski, VT, USA) by recording OD660 every 5 min during 12–24 h. Biomass of each aging vial at one age-point was measured as the average reading of OD values at 660 nm from 10 to 30 min in outgrowth curves, and the total biomass production of each media was defined as the mean biomass from day 10 to day 22 (**Figure** **S2**).

After one day incubation of the diluted culture at 4°C, the cells were washed twice with water to remove other nutrients, 5.0 µL of the diluted cells was pipetted into each well of 96-well microplate. One hundredµL of different media was then added to each well. The cell population was monitored with a microplate reader by recording the OD every 5 min at 660 nm (**Figure** **S3**).

### Data analysis

The raw data from the microplate reader were exported to Excel (Microsoft, San Leandro, CA, USA). From the growth curves, the viability of the yeast can be obtained according to our previous report [22]. Survival integral (SI) of each aging culture was defined as the area under the survival curves (AUC) (**Figure** **S1**). For surface-response data analysis, the SAS program automatically provides tools that are appropriate for examining the linear and the quadratic effects, for estimating model parameters, for carrying out an analysis of variance (**Table** **S2**), for fitting models that can be used to find optimal factor settings, and for generating the surface-response plots (**Figure** **3** **and** **4**). Correlation among lifespan, biomass production and viability of day 2 was computed by using the SAS CORR procedure, which can provide Pearson correlation coefficients and associated probabilities (**Table** **2**). The analysis of variance for each set of biological replicates was carried out with the SAS statistical program, and differences between the means of SI for treatments were determined by Duncan’s multiple range test at *P*<0.05.

## Supporting Information

Figure S1 **Survival curves of four yeast strains.** Four yeast strains (WT, *sch9*Δ, *tor1*Δ, and *sir2*Δ) were cultured in 13 media for 22 days. The relative survival of each age-point was shown as the mean within 16 replicates.
(TIF)

Figure S2 **Effect of media nutrients composition on yeast biomass production.** Biomass of each aging vial at one age-point was measured as the average reading of OD values at 660 nm from 10 to 30 min in outgrowth curves. The OD value of The SD medium at day 2 was defined as 100%. Data is shown as the mean within 16 replicates (RSD <10%).
(TIF)

Figure S3 **Different media have little effect on cell growth during lag phase in most yeast strains.** The growth curves show that yeast cells of WT (A), *sir2*Δ (B), *sch9*Δ (C) and *tor1*Δ (D) proliferated well with nutrients available in different media since the lag time (≈ 8 h) of each curve had no significant changes. Yeast cultured in media containing high and balanced AAs, glucose and YNB content produced a higher number of cells. However, *sch9*Δ did not grow well in several media, even in the SD (C). FiveµL of diluted and nutrient free yeast culture (≈ 1×10^4^ cells) was pipetted into each well of 96-well microplate. One hundredµL of different media was then added to each well. The cell population was monitored with a microplate reader by recording the OD every 5 min at 660 nm.
(TIF)

Figure S4 **Representative cell images of the four yeast strains in different media at day 22.** Yeast cells at different aging-points were collected and observed using an optical microscope (Olympus CX31, Tokyo, Japan) with 1000× magnification. *sch9*Δ cells gathered together in response to nutrient imbalance in the media.
(TIF)

Table S1 **Composition of synthetic defined (SD) media used for yeast chronological lifespan analysis.**
(DOC)

Table S2 **The linear and the quadratic parameter estimates for lifespan and biomass production.**
(DOC)

Table S3 **Membership function value f(x) and ranking for the 15 media according to the two criteria of lifespan and biomass in the four strains.**
(DOC)

## References

[1] McCay C, Crowell M, Maynard L (1935) The effect of retarded growth upon the length of life span and upon the ultimate body size: one figure. J Nutri 10: 63. 2520283

[2] Mair W, Dillin A (2008) Aging and survival: the genetics of life span extension by dietary restriction. Annu Rev Biochem 77: 727–754. 18373439 10.1146/annurev.biochem.77.061206.171059

[3] Colman RJ, Anderson RM, Johnson SC, Kastman EK, Kosmatka KJ, et al (2009) Caloric restriction delays disease onset and mortality in rhesus monkeys. Science 325: 201–204. 19590001 10.1126/science.1173635 PMC2812811

[4] Kaeberlein M, Burtner CR, Kennedy BK (2007) Recent developments in yeast aging. PLoS Genet 3: e84. 17530929 10.1371/journal.pgen.0030084 PMC1877880

[5] Jiang JC, Jaruga E, Repnevskaya MV, Jazwinski SM (2000) An intervention resembling caloric restriction prolongs life span and retards aging in yeast. FASEB J 14: 2135–2137. 11024000 10.1096/fj.00-0242fje

[6] Powers RW, 3rd, Kaeberlein M, Caldwell SD, Kennedy BK, Fields S (2006) Extension of chronological life span in yeast by decreased TOR pathway signaling. Genes Dev 20: 174–184. 16418483 10.1101/gad.1381406 PMC1356109

[7] Greer EL, Brunet A (2009) Different dietary restriction regimens extend lifespan by both independent and overlapping genetic pathways in *C. elegans* . Aging Cell 8: 113–127. 19239417 10.1111/j.1474-9726.2009.00459.x PMC2680339

[8] Fanson BG, Weldon CW, Perez-Staples D, Simpson SJ, Taylor PW (2009) Nutrients, not caloric restriction, extend lifespan in Queensland fruit flies (*Bactrocera tryoni*). Aging Cell 8: 514–523. 19558564 10.1111/j.1474-9726.2009.00497.x

[9] Grandison RC, Piper MD, Partridge L (2009) Amino-acid imbalance explains extension of lifespan by dietary restriction in *Drosophila* . Nature 462: 1061–1064. 19956092 10.1038/nature08619 PMC2798000

[10] Lee KP, Simpson SJ, Clissold FJ, Brooks R, Ballard JW, et al (2008) Lifespan and reproduction in *Drosophila*: New insights from nutritional geometry. Proc Natl Acad Sci U S A 105: 2498–2503. 18268352 10.1073/pnas.0710787105 PMC2268165

[11] Ja WW, Carvalho GB, Zid BM, Mak EM, Brummel T, et al (2009) Water- and nutrient-dependent effects of dietary restriction on *Drosophila* lifespan. Proc Natl Acad Sci U S A 106: 18633–18637. 19841272 10.1073/pnas.0908016106 PMC2773996

[12] Zimmerman JA, Malloy V, Krajcik R, Orentreich N (2003) Nutritional control of aging. Exp Gerontol 38: 47–52. 12543260 10.1016/s0531-5565(02)00149-3

[13] Miller RA, Buehner G, Chang Y, Harper JM, Sigler R, et al (2005) Methionine-deficient diet extends mouse lifespan, slows immune and lens aging, alters glucose, T4, IGF-I and insulin levels, and increases hepatocyte MIF levels and stress resistance. Aging Cell 4: 119–125. 15924568 10.1111/j.1474-9726.2005.00152.x PMC7159399

[14] Piper MD, Partridge L, Raubenheimer D, Simpson SJ (2011) Dietary restriction and aging: a unifying perspective. Cell Metab 14: 154–160. 21803286 10.1016/j.cmet.2011.06.013 PMC4445606

[15] Bishop NA, Guarente L (2007) Genetic links between diet and lifespan: shared mechanisms from yeast to humans. Nat Rev Genet 8: 835–844. 17909538 10.1038/nrg2188

[16] Burtner CR, Murakami CJ, Kennedy BK, Kaeberlein M (2009) A molecular mechanism of chronological aging in yeast. Cell Cycle 8: 1256–1270. 19305133 10.4161/cc.8.8.8287 PMC2746416

[17] Murakami CJ, Wall V, Basisty N, Kaeberlein M (2011) Composition and acidification of the culture media influences chronological aging similarly in vineyard and laboratory yeast. PLoS One 6: e24530. 21949725 10.1371/journal.pone.0024530 PMC3176285

[18] Murakami C, Delaney JR, Chou A, Carr D, Schleit J, et al (2012) pH neutralization protects against reduction in replicative lifespan following chronological aging in yeast. Cell Cycle 11: 3087–3096. 22871733 10.4161/cc.21465 PMC3442919

[19] Alvers AL, Fishwick LK, Wood MS, Hu D, Chung HS, et al (2009) Autophagy and amino acid homeostasis are required for chronological longevity in *Saccharomyces cerevisiae* . Aging Cell 8: 353–369. 19302372 10.1111/j.1474-9726.2009.00469.x PMC2802268

[20] Skorupa DA, Dervisefendic A, Zwiener J, Pletcher SD (2008) Dietary composition specifies consumption, obesity, and lifespan in *Drosophila melanogaster* . Aging Cell 7: 478–490. 18485125 10.1111/j.1474-9726.2008.00400.x PMC2574586

[21] Baş D, Boyacı İH (2007) Modeling and optimization I: Usability of response surface methodology. J Food Eng 78: 836–845.

[22] Wu Z, Song L, Liu SQ, Huang D (2011) A high throughput screening assay for determination of chronological lifespan of yeast. Exp Gerontol 46: 915–922. 21871551 10.1016/j.exger.2011.08.002

[23] Fontana L, Partridge L, Longo VD (2010) Extending healthy life span—from yeast to humans. Science 328: 321–326. 20395504 10.1126/science.1172539 PMC3607354

[24] Kaeberlein M, Steffen KK, Hu D, Dang N, Kerr EO, et al.. (2006) Comment on "HST2 mediates SIR2-independent life-span extension by calorie restriction". Science 312: 1312; author reply 1312. 10.1126/science.1124608 16741098

[25] Smith DL Jr, McClure JM, Matecic M, Smith JS (2007) Calorie restriction extends the chronological lifespan of *Saccharomyces cerevisiae* independently of the Sirtuins. Aging Cell 6: 649–662. 17711561 10.1111/j.1474-9726.2007.00326.x

[26] Lamming DW, Latorre-Esteves M, Medvedik O, Wong SN, Tsang FA, et al.. (2006) Response to comment on "HST2 mediates SIR2-independent life-span extension by calorie restriction". Science 312. 10.1126/science.1113611 16051752

[27] Goldberg AA, Bourque SD, Kyryakov P, Gregg C, Boukh-Viner T, et al (2009) Effect of calorie restriction on the metabolic history of chronologically aging yeast. Exp Gerontol 44: 555–571. 19539741 10.1016/j.exger.2009.06.001

[28] Aragon AD, Rodriguez AL, Meirelles O, Roy S, Davidson GS, et al (2008) Characterization of differentiated quiescent and nonquiescent cells in yeast stationary-phase cultures. Mol Biol Cell 19: 1271–1280. 18199684 10.1091/mbc.E07-07-0666 PMC2262958

[29] Fabrizio P, Battistella L, Vardavas R, Gattazzo C, Liou LL, et al (2004) Superoxide is a mediator of an altruistic aging program in *Saccharomyces cerevisiae* . J Cell Biol 166: 1055–1067. 15452146 10.1083/jcb.200404002 PMC2172019

[30] Kaeberlein M (2010) Lessons on longevity from budding yeast. Nature 464: 513–519. 20336133 10.1038/nature08981 PMC3696189

[31] Guarente L (2011) Franklin H. Epstein Lecture: Sirtuins, aging, and medicine. N Engl J Med 364: 2235–2244. 21651395 10.1056/NEJMra1100831

[32] Haigis MC, Sinclair DA (2010) Mammalian sirtuins: biological insights and disease relevance. Annu Rev Pathol 5: 253–295. 20078221 10.1146/annurev.pathol.4.110807.092250 PMC2866163

[33] Lombard DB, Pletcher SD, Canto C, Auwerx J (2011) Aging: longevity hits a roadblock. Nature 477: 410–411. 21938058 10.1038/477410a

[34] Fabrizio P, Hoon S, Shamalnasab M, Galbani A, Wei M, et al (2010) Genome-wide screen in *Saccharomyces cerevisiae* identifies vacuolar protein sorting, autophagy, biosynthetic, and tRNA methylation genes involved in life span regulation. PLoS Genet 6: e1001024. 20657825 10.1371/journal.pgen.1001024 PMC2904796

[35] Fabrizio P, Longo VD (2003) The chronological life span of *Saccharomyces cerevisiae* . Aging Cell 2: 73–81. 12882320 10.1046/j.1474-9728.2003.00033.x

[36] Thomas KC, Hynes SH, Ingledew WM (2002) Influence of media buffering capacity on inhibition of *Saccharomyces cerevisiae* growth by acetic and lactic acids. Appl Environ Microbiol 68: 1616–1623. 11916676 10.1128/AEM.68.4.1616-1623.2002 PMC123831

[37] Meydani M (2001) Nutrition interventions in aging and age-associated disease. Ann N Y Acad Sci 928: 226–235. 11795514 10.1111/j.1749-6632.2001.tb05652.x

[38] Longo VD, Shadel GS, Kaeberlein M, Kennedy B (2012) Replicative and chronological aging in *Saccharomyces cerevisiae* . Cell Metab 16: 18–31. 22768836 10.1016/j.cmet.2012.06.002 PMC3392685

[39] Murakami CJ, Burtner CR, Kennedy BK, Kaeberlein M (2008) A method for high-throughput quantitative analysis of yeast chronological life span. J Gerontol A Biol Sci Med Sci 63: 113–121. 18314444 10.1093/gerona/63.2.113

[40] Allen C, Buttner S, Aragon AD, Thomas JA, Meirelles O, et al (2006) Isolation of quiescent and nonquiescent cells from yeast stationary-phase cultures. J Cell Biol 174: 89–100. 16818721 10.1083/jcb.200604072 PMC2064167

[41] Minois N, Lagona F, Frajnt M, Vaupel JW (2009) Plasticity of death rates in stationary phase in *Saccharomyces cerevisiae* . Aging Cell 8: 36–44. 19053971 10.1111/j.1474-9726.2008.00446.x

[42] Koc A, Gasch AP, Rutherford JC, Kim HY, Gladyshev VN (2004) Methionine sulfoxide reductase regulation of yeast lifespan reveals reactive oxygen species-dependent and -independent components of aging. Proc Natl Acad Sci U S A 101: 7999–8004. 15141092 10.1073/pnas.0307929101 PMC419546

[43] Malloy VL, Krajcik RA, Bailey SJ, Hristopoulos G, Plummer JD, et al (2006) Methionine restriction decreases visceral fat mass and preserves insulin action in aging male Fischer 344 rats independent of energy restriction. Aging Cell 5: 305–314. 16800846 10.1111/j.1474-9726.2006.00220.x

[44] Elshorbagy AK, Valdivia-Garcia M, Refsum H, Smith AD, Mattocks DA, et al (2010) Sulfur amino acids in methionine-restricted rats: hyperhomocysteinemia. Nutriti 26: 1201–1204. 10.1016/j.nut.2009.09.017 20080389

[45] Urban J, Soulard A, Huber A, Lippman S, Mukhopadhyay D, et al (2007) Sch9 is a major target of TORC1 in *Saccharomyces cerevisiae* . Mol Cell 26: 663–674. 17560372 10.1016/j.molcel.2007.04.020

[46] Jorgensen P, Nishikawa JL, Breitkreutz BJ, Tyers M (2002) Systematic identification of pathways that couple cell growth and division in yeast. Science 297: 395–400. 12089449 10.1126/science.1070850

[47] Kaeberlein M, Powers RW, 3rd, Steffen KK, Westman EA, Hu D, et al (2005) Regulation of yeast replicative life span by TOR and Sch9 in response to nutrients. Science 310: 1193–1196. 16293764 10.1126/science.1115535

[48] Fabrizio P, Pozza F, Pletcher SD, Gendron CM, Longo VD (2001) Regulation of longevity and stress resistance by Sch9 in yeast. Science 292: 288–290. 11292860 10.1126/science.1059497

[49] Mieulet V, Yan L, Lamb RF (2009) Signalling by amino acid nutrients: mTOR and beyond. Amino Acids 37: 67–67.

[50] Huber A, Bodenmiller B, Uotila A, Stahl M, Wanka S, et al (2009) Characterization of the rapamycin-sensitive phosphoproteome reveals that Sch9 is a central coordinator of protein synthesis. Genes Dev 23: 1929–1943. 19684113 10.1101/gad.532109 PMC2725941

[51] Huber A, French SL, Tekotte H, Yerlikaya S, Stahl M, et al (2011) Sch9 regulates ribosome biogenesis via Stb3, Dot6 and Tod6 and the histone deacetylase complex RPD3L. Embo J 30: 3052–3064. 21730963 10.1038/emboj.2011.221 PMC3160192

