# Supplementary figures and images for: Dietary Restriction Depends on Nutrient Composition to Extend Chronological Lifespan in Budding Yeast Saccharomyces cerevisiae
Source: PLoS One. 2013 May 17;8(5):e64448. doi: 10.1371/journal.pone.0064448 (PMC3656888; doi:10.1371/journal.pone.0064448)

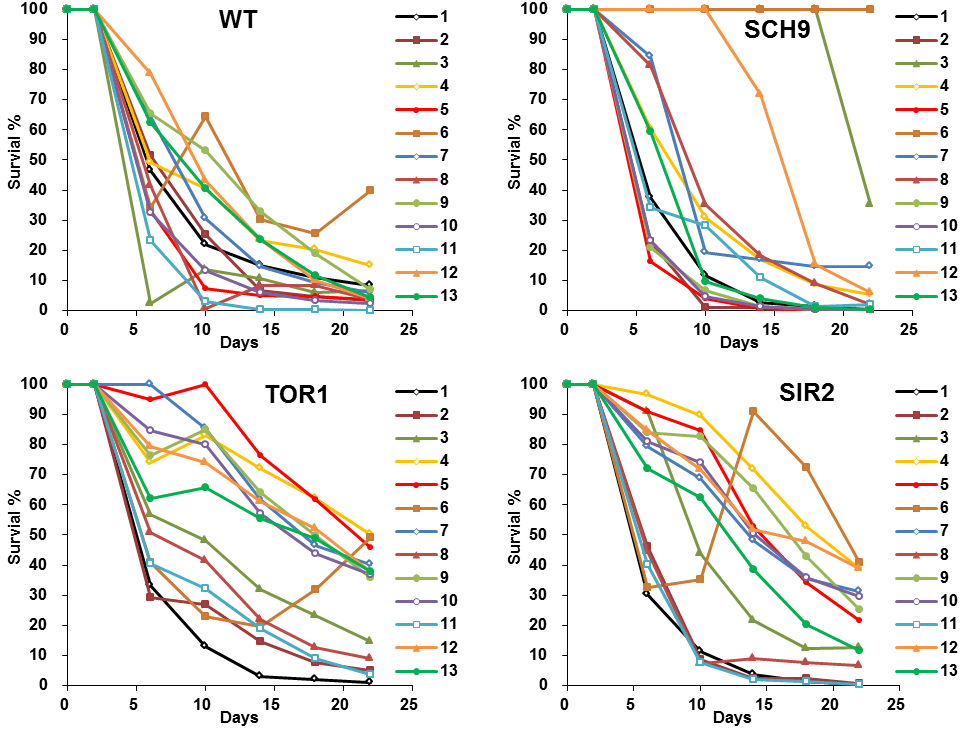

Supplement: Figure S1 — Survival curves of four yeast strains. Four yeast strains (WT, sch9Δ, tor1Δ, and sir2Δ) were cultured in 13 media for 22 days. The relative survival of each age-point was shown as the mean within 16 replicates. (TIF) [file pone.0064448.s001.tif]

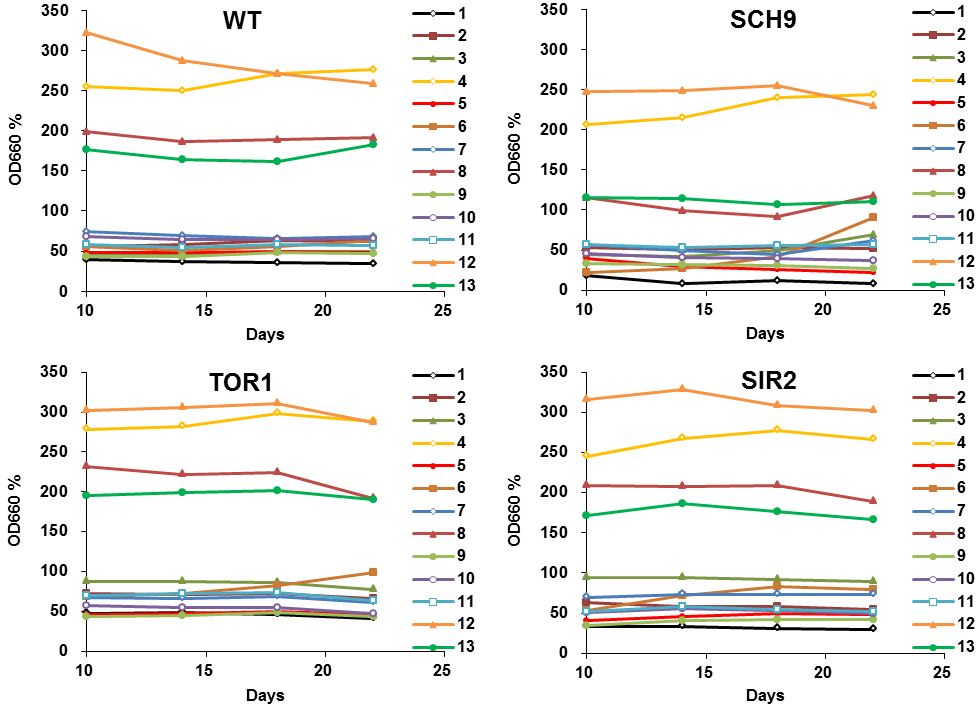

Supplement: Figure S2 — Effect of media nutrients composition on yeast biomass production. Biomass of each aging vial at one age-point was measured as the average reading of OD values at 660 nm from 10 to 30 min in outgrowth curves. The OD value of The SD medium at day 2 was defined as 100%. Data is shown as the mean within 16 replicates (RSD <10%). (TIF) [file pone.0064448.s002.tif]

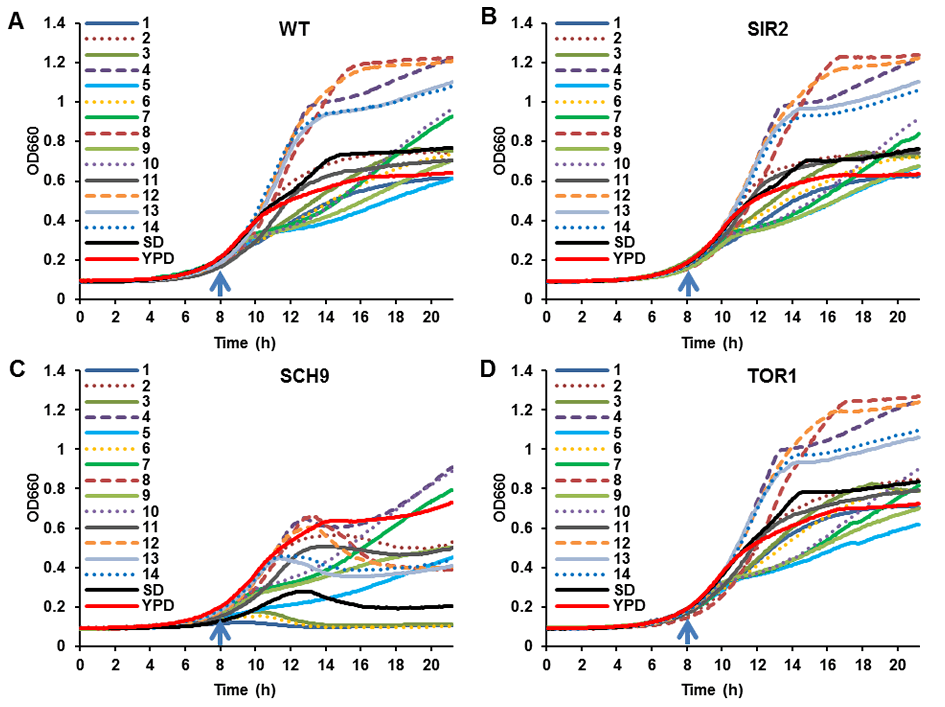

Supplement: Figure S3 — Different media have little effect on cell growth during lag phase in most yeast strains. The growth curves show that yeast cells of WT (A), sir2Δ (B), sch9Δ (C) and tor1Δ (D) proliferated well with nutrients available in different media since the lag time (≈ 8 h) of each curve had no significant changes. Yeast cultured in media containing high and balanced AAs, glucose and YNB content produced a higher number of cells. However, sch9Δ did not grow well in several media, even in the SD (C). FiveµL of diluted and nutrient free yeast culture (≈ 1×10^4^ cells) was pipetted into each well of 96-well microplate. One hundredµL of different media was then added to each well. The cell population was monitored with a microplate reader by recording the OD every 5 min at 660 nm. (TIF) [file pone.0064448.s003.tif]

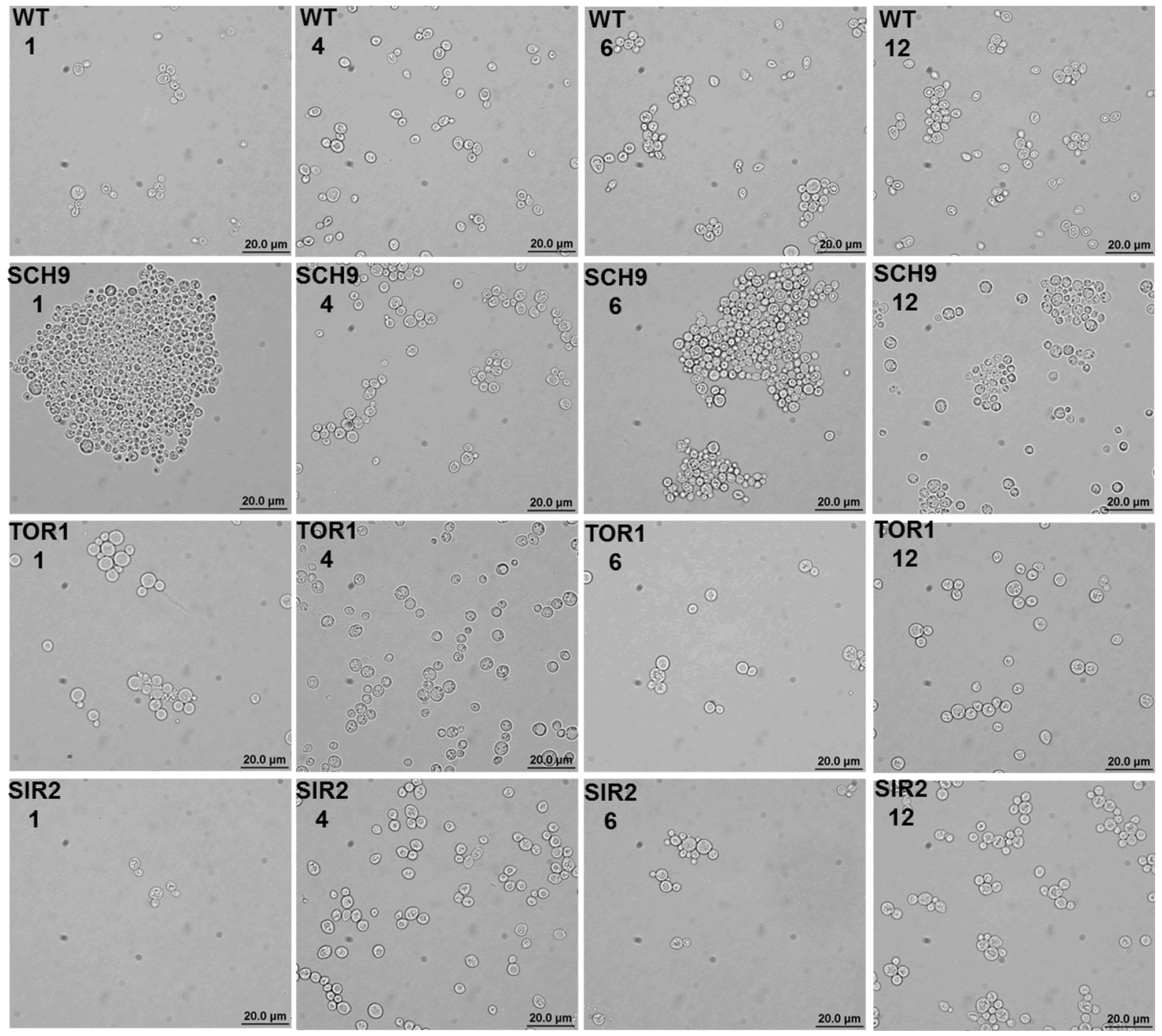

Supplement: Figure S4 — Representative cell images of the four yeast strains in different media at day 22. Yeast cells at different aging-points were collected and observed using an optical microscope (Olympus CX31, Tokyo, Japan) with 1000× magnification. sch9Δ cells gathered together in response to nutrient imbalance in the media. (TIF) [file pone.0064448.s004.tif]
