# Supplementary material for: Dietary Restriction Depends on Nutrient Composition to Extend Chronological Lifespan in Budding Yeast Saccharomyces cerevisiae
Source: PLoS One. 2013 May 17;8(5):e64448. doi: 10.1371/journal.pone.0064448 (PMC3656888; doi:10.1371/journal.pone.0064448)
Supplement: Table S1 — Composition of synthetic defined (SD) media used for yeast chronological lifespan analysis. (DOC) [file pone.0064448.s005.doc]

**Table S1. Composition of synthetic defined (SD) medium used for yeast** **chronological life span analysis**

| Component | Concentration |
| --- | --- |
| **Glucose** | 20 g/L |
| **Yeast Nitrogen Base (-AA/-AS)** | 1.7 g/L |
| *Potassium phosphate* | *1 g/L* |
| *Magnesium sulfate* | *500 mg/L* |
| *Sodium chloride* | *100 mg/L* |
| *Calcium chloride* | *100 mg/L* |
| *Biotin* | *0.002 mg/L* |
| *Pantothenate* | *0.4 mg/L* |
| *Folate* | *0.002 mg/L* |
| *Inositol* | *2 mg/L* |
| *Niacin* | *0.4 mg/L* |
| *PABA* | *0.2 mg/L* |
| *Pyridoxine, HCl* | *0.4 mg/L* |
| *Riboflavin* | *0.2 mg/L* |
| *Thiamine, HCl* | *0.4 mg/L* |
| *Riboflavin* | *0.2 mg/L* |
| *Thiamine, HCl* | *0.4 mg/L* |
| *Boric acid* | *0.5 mg/L* |
| *Copper sulfate* | *0.04 mg/L* |
| *Potassium iodide* | *0.1 mg/L* |
| *Ferric chloride* | *0.2 mg/L* |
| *Manganese sulfate* | *0.4 mg/L* |
| *Sodium molybdate* | *0.2 mg/L* |
| *Zinc sulfate* | *0.4 mg/L* |
| Ammonium sulfate | 5 g/L |
| **Amino acids (1×)** |  |
| *Adenine* | *80 mg/L* |
| *Uracil* | *100 mg/L* |
| *L-arginine* | *40 mg/L* |
| *L-aspartate* | *100 mg/L* |
| *L-glutamate* | *100 mg/L* |
| *L-histidine* | *100 mg/L* |
| *L-leucine* | *300 mg/L* |
| *L-lysine* | *150 mg/L* |
| *L-methionine* | *80 mg/L* |
| *L-phenylalanine* | *50 mg/L* |
| *L-serine* | *400 mg/L* |
| *L-threonine* | *200 mg/L* |
| *L-tryptophan* | *200 mg/L* |
| *L-tyrosine* | *40 mg/L* |
| *L-valine* | *150 mg/L* |
| *L-isoleucine* | *60 mg/L* |
