# Supplementary material for: Dietary Restriction Depends on Nutrient Composition to Extend Chronological Lifespan in Budding Yeast Saccharomyces cerevisiae
Source: PLoS One. 2013 May 17;8(5):e64448. doi: 10.1371/journal.pone.0064448 (PMC3656888; doi:10.1371/journal.pone.0064448)
Supplement: Table S2 — The linear and the quadratic parameter estimates for lifespan and biomass production. (DOC) [file pone.0064448.s006.doc]

**Table S2. The linear and the quadratic parameter estimates for lifespan and biomass production**

| Term | | WT | | |  | SCH9 | | |  | TOR1 | | |  | SIR2 | | |
| --- | --- | --- | --- | --- | --- | --- | --- | --- | --- | --- | --- | --- | --- | --- | --- | --- |
|  |  | Lifespan |  | Biomass |  | Lifespan |  | Biomass |  | Lifespan |  | Biomass |  | Lifespan |  | Biomass |
| AA | Estimate a | 0.63 |  | 57.85 |  | 538.88 |  | 51.69 |  | 262.63 |  | 61.98 |  | 294.50 |  | 67.28 |
|  | P > |t| | 0.9880 |  | 0.0007 |  | <.0001 |  | 0.0070 |  | 0.0233 |  | 0.0002 |  | 0.0276 |  | 0.0002 |
| YNB | Estimate | 26.13 |  | 49.38 |  | -415.63 |  | 37.61 |  | 75.75 |  | 47.84 |  | -18.75 |  | 44.61 |
|  | P > |t| | 0.5394 |  | 0.0014 |  | 0.0001 |  | 0.0239 |  | 0.3947 |  | 0.0007 |  | 0.8525 |  | 0.0014 |
| GLU | Estimate | 7.75 |  | 45.00 |  | 586.00 |  | 37.25 |  | -309.88 |  | 57.29 |  | -207.00 |  | 54.21 |
|  | P > |t| | 0.8529 |  | 0.0021 |  | <.0001 |  | 0.0248 |  | 0.0125 |  | 0.0003 |  | 0.0830 |  | 0.0006 |
| AA x AA | Estimate | -100.25 |  | -26.99 |  | 107.58 |  | 0.00 |  | -194.88 |  | -30.65 |  | -94.29 |  | -23.00 |
|  | P > |t| | 0.1468 |  | 0.0636 |  | 0.1241 |  | 0.9998 |  | 0.1647 |  | 0.0224 |  | 0.5332 |  | 0.0773 |
| AA x YNB | Estimate | 113.00 |  | 48.08 |  | -417.00 |  | 33.88 |  | 119.00 |  | 44.13 |  | 142.25 |  | 36.33 |
|  | P > |t| | 0.1002 |  | 0.0070 |  | 0.0007 |  | 0.0969 |  | 0.3486 |  | 0.0045 |  | 0.3416 |  | 0.0148 |
| AA x GLU | Estimate | 247.75 |  | 51.88 |  | 472.75 |  | 44.95 |  | 192.25 |  | 55.78 |  | 255.75 |  | 61.73 |
|  | P > |t| | 0.0069 |  | 0.0051 |  | 0.0004 |  | 0.0424 |  | 0.1557 |  | 0.0016 |  | 0.1176 |  | 0.0016 |
| YNB x YNB | Estimate | -105.75 |  | -48.89 |  | 357.08 |  | -34.80 |  | -187.63 |  | -45.33 |  | -120.29 |  | -39.63 |
|  | P > |t| | 0.1300 |  | 0.0077 |  | 0.0017 |  | 0.1003 |  | 0.1781 |  | 0.0048 |  | 0.4324 |  | 0.0123 |
| YNB x GLU | Estimate | -183.75 |  | 28.63 |  | -601.25 |  | 9.20 |  | 10.50 |  | 29.40 |  | -152.75 |  | 26.35 |
|  | P > |t| | 0.0221 |  | 0.0471 |  | 0.0001 |  | 0.6035 |  | 0.9309 |  | 0.0225 |  | 0.3106 |  | 0.0457 |
| GLU x GLU | Estimate | -55.50 |  | -37.39 |  | 278.83 |  | -27.02 |  | 162.13 |  | -49.93 |  | 138.21 |  | -36.23 |
|  | P > |t| | 0.3857 |  | 0.0217 |  | 0.0049 |  | 0.1788 |  | 0.2339 |  | 0.0032 |  | 0.3719 |  | 0.0174 |
