# Supplementary material for: Dietary Restriction Depends on Nutrient Composition to Extend Chronological Lifespan in Budding Yeast Saccharomyces cerevisiae
Source: PLoS One. 2013 May 17;8(5):e64448. doi: 10.1371/journal.pone.0064448 (PMC3656888; doi:10.1371/journal.pone.0064448)
Supplement: Table S3 — Membership function value f(x) and ranking for the 15 media according to the two criteria of lifespan and biomass in the four strains. (DOC) [file pone.0064448.s007.doc]

**Table S3. Membership function value f(x) and ranking for the 15 media according the two criteria of lifespan and biomass in the four yeast strains**

|  | WT | |  | SCH9 | |  | TOR1 | |  | SIR2 | |  |  |  |
| --- | --- | --- | --- | --- | --- | --- | --- | --- | --- | --- | --- | --- | --- | --- |
| Run | lifespan | Biomass |  | lifespan | Biomass |  | lifespan | Biomass |  | lifespan | Biomass |  | Mean | Ranking |
| 1 | 0.48 | 0.00 |  | 0.05 | 0.00 |  | 0.00 | 0.00 |  | 0.00 | 0.00 |  | 0.07 | 15 |
| 2 | 0.43 | 0.09 |  | 0.01 | 0.17 |  | 0.10 | 0.10 |  | 0.05 | 0.09 |  | 0.13 | 13 |
| 3 | 0.06 | 0.04 |  | 0.90 | 0.17 |  | 0.38 | 0.16 |  | 0.45 | 0.21 |  | 0.30 | 10 |
| 4 | 0.77 | 0.91 |  | 0.16 | 0.92 |  | 0.86 | 0.94 |  | 1.00 | 0.82 |  | 0.80 | 2 |
| 5 | 0.17 | 0.05 |  | 0.00 | 0.07 |  | 1.00 | 0.01 |  | 0.80 | 0.05 |  | 0.27 | 11 |
| 6 | 1.00 | 0.08 |  | 1.00 | 0.14 |  | 0.29 | 0.14 |  | 0.72 | 0.14 |  | 0.44 | 6 |
| 7 | 0.64 | 0.13 |  | 0.20 | 0.18 |  | 0.94 | 0.08 |  | 0.71 | 0.14 |  | 0.38 | 8 |
| 8 | 0.23 | 0.62 |  | 0.20 | 0.40 |  | 0.26 | 0.67 |  | 0.09 | 0.61 |  | 0.39 | 7 |
| 9 | 0.99 | 0.04 |  | 0.01 | 0.08 |  | 0.78 | 0.00 |  | 0.85 | 0.03 |  | 0.35 | 9 |
| 10 | 0.20 | 0.12 |  | 0.01 | 0.12 |  | 0.75 | 0.03 |  | 0.74 | 0.07 |  | 0.26 | 12 |
| 11 | 0.00 | 0.08 |  | 0.09 | 0.19 |  | 0.17 | 0.10 |  | 0.02 | 0.08 |  | 0.09 | 14 |
| 12 | 0.89 | 1.00 |  | 0.87 | 1.00 |  | 0.76 | 1.00 |  | 0.81 | 1.00 |  | 0.92 | 1 |
| 13 | 0.77 | 0.54 |  | 0.09 | 0.43 |  | 0.65 | 0.59 |  | 0.54 | 0.51 |  | 0.51 | 5 |
| 14 | 0.80 | 0.60 |  | 0.09 | 0.49 |  | 0.64 | 0.60 |  | 0.61 | 0.51 |  | 0.54 | 3 |
| 15 | 0.79 | 0.56 |  | 0.09 | 0.47 |  | 0.64 | 0.60 |  | 0.55 | 0.49 |  | 0.52 | 4 |

f(x) = (x – x_min_)/(x_max_ – x_min_), x is the lifespan and biomass among 15 runs respectively, ranking based on means of f(x) values of lifespan and biomass in three strains, the number of ranking is smaller, indicating the medium for yeast culture is better for longevity and biomass production.
